# Distinct Growth Responses of Tundra Soil Bacteria to Short-Term and Long-Term Warming

**DOI:** 10.1128/aem.01543-22

**Published:** 2023-02-27

**Authors:** Jeffrey R. Propster, Egbert Schwartz, Michaela Hayer, Samantha Miller, Victoria Monsaint-Queeney, Benjamin J. Koch, Ember M. Morrissey, Michelle C. Mack, Bruce A. Hungate

**Affiliations:** a Center for Ecosystem Science and Society, Northern Arizona University, Flagstaff, Arizona, USA; b Department of Biological Sciences, Northern Arizona University, Flagstaff, Arizona, USA; c Division of Plant and Soil Sciences, West Virginia University, Morgantown, West Virginia, USA; Colorado School of Mines

**Keywords:** Arctic tundra, Toolik LTER, climate change, field qSIP, phylogenetic signal, soil bacterial growth

## Abstract

Increases in Arctic temperatures have thawed permafrost and accelerated tundra soil microbial activity, releasing greenhouse gases that amplify climate warming. Warming over time has also accelerated shrub encroachment in the tundra, altering plant input abundance and quality, and causing further changes to soil microbial processes. To better understand the effects of increased temperature and the accumulated effects of climate change on soil bacterial activity, we quantified the growth responses of individual bacterial taxa to short-term warming (3 months) and long-term warming (29 years) in moist acidic tussock tundra. Intact soil was assayed in the field for 30 days using ^18^O-labeled water, from which taxon-specific rates of ^18^O incorporation into DNA were estimated as a proxy for growth. Experimental treatments warmed the soil by approximately 1.5°C. Short-term warming increased average relative growth rates across the assemblage by 36%, and this increase was attributable to emergent growing taxa not detected in other treatments that doubled the diversity of growing bacteria. However, long-term warming increased average relative growth rates by 151%, and this was largely attributable to taxa that co-occurred in the ambient temperature controls. There was also coherence in relative growth rates within broad taxonomic levels with orders tending to have similar growth rates in all treatments. Growth responses tended to be neutral in short-term warming and positive in long-term warming for most taxa and phylogenetic groups co-occurring across treatments regardless of phylogeny. Taken together, growing bacteria responded distinctly to short-term and long-term warming, and taxa growing in each treatment exhibited deep phylogenetic organization.

**IMPORTANCE** Soil carbon stocks in the tundra and underlying permafrost have become increasingly vulnerable to microbial decomposition due to climate change. The microbial responses to Arctic warming must be understood in order to predict the effects of future microbial activity on carbon balance in a warming Arctic. In response to our warming treatments, tundra soil bacteria grew faster, consistent with increased rates of decomposition and carbon flux to the atmosphere. Our findings suggest that bacterial growth rates may continue to increase in the coming decades as faster growth is driven by the accumulated effects of long-term warming. Observed phylogenetic organization of bacterial growth rates may also permit taxonomy-based predictions of bacterial responses to climate change and inclusion into ecosystem models.

## INTRODUCTION

Arctic tundra ecosystems harbor the largest terrestrial carbon stock frozen in underlying layers of permafrost (1, 2). Climate change is warming the Arctic at twice the rate of lower latitudes, with an observed increase of 0.75°C in just the last decade (3, 4). These increasing temperatures are thawing organic carbon within permafrost, making it vulnerable to microbial decomposition and shifting Arctic tundra from a net carbon sink to a net carbon source (5, 6). Increased flux of greenhouse gases from permafrost is predicted to exacerbate the soil-climate feedback (7–9), but this increase is partially offset by subsequent ecosystem changes in the annually thawed active layer. In the short term, warming and increased microbial activity drive a complex cascade of interacting effects on tundra nutrient cycling, plant communities, and carbon balance (10–12). As soils warm, microbial communities increase rates of decomposition and nutrient cycling (13–15). More available soil nutrients combined with warmer summers (16) and an extended growing season (3) stimulate plant productivity, particularly of woody shrubs (6, 17). Shrub encroachment has been observed across the Arctic (18, 19) and is associated with increased input of litter and roots of differing chemistry that alters rates of decomposition (17, 20, 21). The net effects of long-term warming increase carbon storage in plant biomass while reducing soil carbon stock over time (10, 14, 22), depending on the tundra type (23, 24).

Understanding how soil microbial activity changes in response to the direct effects of increased temperatures and the indirect effects of climate change accumulated over time (shrub encroachment, altered litter inputs, etc.) is essential to disentangling the underlying mechanisms of microbial carbon flux from tundra ecosystems to the atmosphere. In general, warming increases tundra microbial activity as measured by the increases in bulk soil respiration (25, 26), carbon and nitrogen cycling enzymes (13, 27, 28), and functional gene abundances (14, 15). Active populations of tundra microbial communities have also been identified using DNA stable isotope probing where the DNA of active members of the tundra microbial community is isotopically labeled based on their assimilation of substrates containing ^13^C (29–31) or ^15^N (32). However, adding an energy source or nutrient can introduce a fertilization artifact, and such isotopic labeling is limited to organisms that assimilate that substrate. Quantitative stable isotope probing (qSIP) using ^18^O water is an effective technique to measure the growth rate of every member of the microbial community with no disturbance other than the addition of water. In the presence of ^18^O-water, replicating populations of microorganisms incorporate ^18^O into their genomes in proportion to their growth rate (33). ^18^O-water is theoretically a universal substrate, meaning all growing organisms can potentially incorporate the label (34). This technique has also been recently implemented in the field to measure growth of intact prokaryotic soil communities (35). Here, we investigated the effects of experimental warming on bacterial growth rates in Arctic tundra soils.

The complexity of microbial soil communities can make interpretations and subsequent predictions of their response to climate change challenging. However, this effort can be simplified by grouping microbes with shared functional traits. Many microbial traits are phylogenetically conserved, meaning that a trait is nonrandomly distributed across a phylogenetic tree, and the complexity of traits influences the level at which they are phylogenetically conserved (36, 37). For example, decomposition of a specific substrate within soil organic matter is performed by bacterial taxa that have functional genes to degrade and metabolize that particular substrate, and the number of genes required for that functional trait influences its depth of phylogenetic conservation (38). Simple carbon substrates such as glucose require fewer genes to be assimilated than more recalcitrant substrates like cellulose; consequently, cellulose assimilation is a functional trait that is predicted to be more deeply phylogenetically conserved (39). Microbial responses to environmental changes are also traits that are conserved within phylogenetic groups. How a soil bacterial population responds to ecosystem changes such as drying-rewetting (40, 41), nutrient addition (42), and climate change (37, 43) all exhibit phylogenetic organization. However, no study has investigated the phylogenetic organization of the response of tundra soil bacteria to climate change. Such responses are typically evaluated as positive or negative changes in relative abundance (38, 44), but bacterial growth responses as measured by stable isotope incorporation into DNA have also been found to be phylogenetically conserved (45–47). Growth rate is an emergent trait that is influenced by numerous genes and genomic characteristics and may therefore exhibit deep phylogenetic conservation (45). However, less complex traits with possible links to tundra warming responses such as substrate assimilation (39) and temperature optima (38) have been found to exhibit phylogenetic conservation at taxonomic levels as low as species. Identifying how individual bacterial taxa and cohesive phylogenetic groups are affected by climate change will improve our ability to predict future ecosystem soil process rates that will influence the magnitude of Arctic soil-climate feedbacks.

To this end, we investigated the effects of short-term and long-term warming on bacterial growth in moist acidic tussock tundra plots that were warmed via plastic greenhouse for 29 years resulting in shrub encroachment, increased primary productivity, altered soil carbon and nitrogen cycling, and shifts in microbial communities (24, 48, 49). Bacterial growth rates in these decadal-scale warming experiments may be predominantly influenced by these indirect effects of warming rather than by increased temperature alone. However, shorter-term responses to warming are more likely to be influenced by the direct, thermal effects as indirect effects have had little time to accumulate. Such a short-term warming treatment was achieved by transplanting intact soil and plant blocks from the control plots into the long-term warming plots, thereby exposing the control microbial community to 3 months of warming. To account for the effects of transplant, similar tundra blocks were cut out of each of the control and long-term warming plot and immediately placed back into their plot of origin. qSIP assays were performed directly in each block using ^18^O incorporation into a taxon’s DNA as a proxy for growth (50, 51). A portion of the environmental water was removed from intact soil and replaced with ^18^O-water, then soil was placed back into the field where it was exposed to environmental conditions for 30 days. We also assessed the phylogenetic organization of tundra bacterial relative growth rates and growth responses to short-term and long-term warming. We hypothesized (i) that bacterial communities would have higher relative growth rates (i.e., ^18^O incorporation) in both warmed treatments, but each treatment would stimulate positive growth responses of different bacterial taxa; short-term warming would elicit a response of taxa that are more sensitive to temperature increases, while long-term warming would stimulate the growth of taxa that are more associated with the accumulated indirect effects of increased temperatures; and (ii) that relative growth rates and growth responses would exhibit phylogenetic organization.

## RESULTS

**FIG 1:**
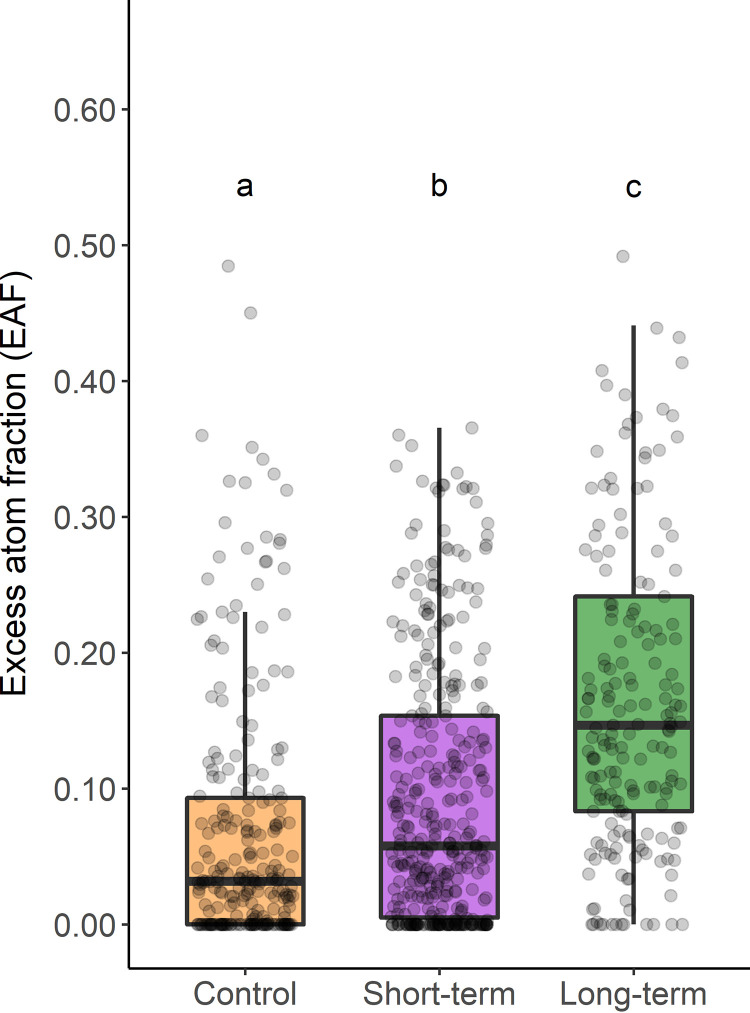
Boxplots of relative growth rates (i.e., excess atom fraction [EAF]) of all bacterial taxa in each treatment. (a to c) Letters indicate significantly different means according to Tukey’s honest significant difference (HSD) test (*P* < 0.001).

The plastic greenhouses increased average soil temperature during the experiment by approximately 1.5°C according to a contemporaneous study (49). As hypothesized, both short-term warming (3 months) and long-term warming (29 years) increased mean relative growth rate of bacteria as measured by ^18^O incorporation (i.e., excess atom fraction [EAF]), and all means were significantly different according to Tukey’s honest significant difference (HSD) *post hoc* comparison (analysis of variance [ANOVA], *F* = 58.6, *P* < 0.0001). The mean ^18^O assimilation (reported as excess atom fraction ^18^O, or EAF) in short-term warming (0.095 ± 0.005) was 36% greater than the unwarmed control (0.070 ± 0.006), and the mean EAF of long-term warming (0.176 ± 0.009) was 151% (or approximately 2.5-fold) greater than the control (Fig. 1). Converted to the Q_10_ temperature sensitivity metric, the growth response to short-term warming corresponds to a Q_10_ of 6.83, and the growth response to long-term warming corresponds to a Q_10_ of 315.

**FIG 2:**
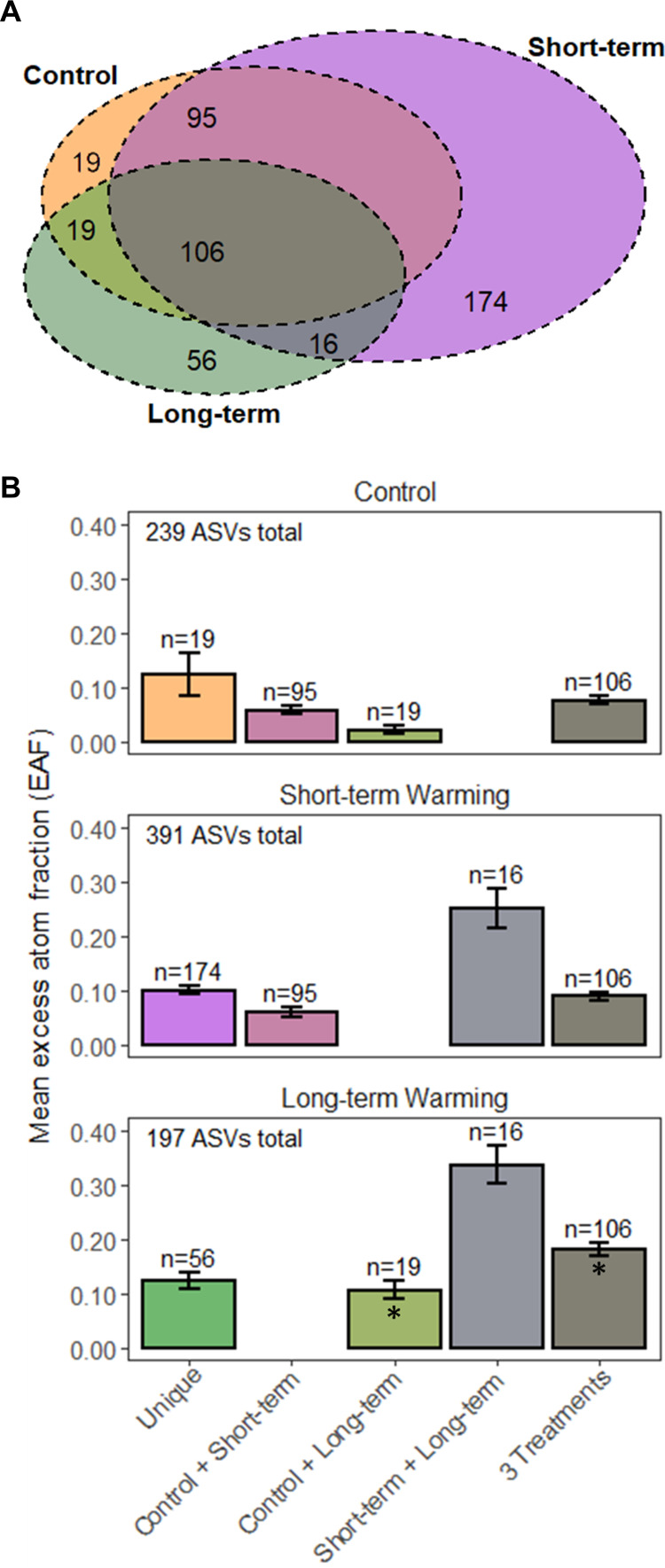
(A) Weighted Venn diagram of taxa occurring across treatments (A). (B) Mean relative growth rate (i.e., excess atom fraction [EAF]) of taxa from each portion of the Venn diagram in each treatment. Bar colors match corresponding regions of the Venn diagram. Asterisks indicate means that were significantly greater in the long-term warming treatment according to either a Student’s *t* test (control + short-term, *P* < 0.001) or Tukey’s HSD test (three treatments, *P* < 0.001). There were no statistically significant differences between short-term warming and control. ASV, amplicon sequence variant.

Short-term warming increased the number of growing bacterial taxa by 64%, but long-term warming decreased total growing bacterial taxa by 18% (Fig. 2A). Considering the portion of taxa shared across treatments and the mean relative growth rates of each shared category (Fig. 2B), short-term warming did not significantly increase the average relative growth rates of taxa that were shared with the control (i.e., control + short-term, three treatments). In contrast, long-term warming significantly increased the relative growth rates of taxa shared with the control (control + short-term, *P* < 0.0013; three treatments, *P* < 0.001). Growing taxa unique to each treatment had similar mean relative growth rates, but short-term warming had many more unique growing taxa (*n* = 174) than long-term warming (*n* = 19) and the control (*n* = 56).

**FIG 3:**
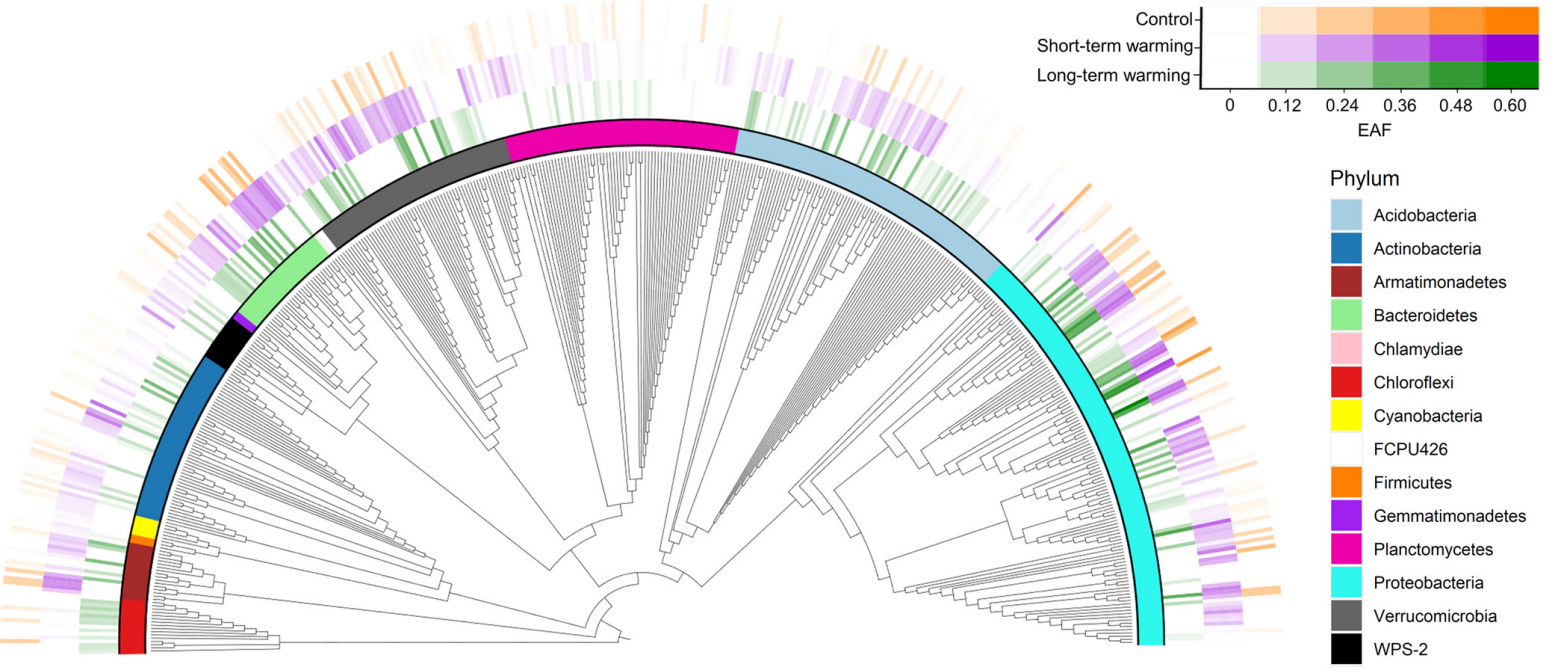
Phylogenetic tree of all taxa with measurable bacterial ^18^O incorporation (i.e., excess atom fraction [EAF]). Darker colors indicate a greater EAF.

We detected 540 taxa with measurable growth across all treatments spanning 13 phyla (Fig. 3). Short-term warming resulted in only two taxa responding positively to warming (i.e., change in ^18^O incorporation compared to control; positive ΔEAF) and one taxon responding negatively, as indicated by confidence intervals not crossing zero (Fig. S1). Only one taxon had a significantly negative growth response in the long-term warming, but 35 taxa spanning 11 phyla had significantly positive responses.

Bacterial growth rates exhibited phylogenetic organization, whereas growth responses to warming did not (Table 1). Incorporation of ^18^O in all treatments exhibited a significant phylogenetic signal for the control, short-term, and long-term warming treatments according to Pagel’s λ (λ = 0.968 and *P* < 0.001; λ = 0.943 and *P* < 0.001; and λ = 0.774 and *P* < 0.001, respectively). Blomberg’s *K* was statistically significant for the control (*K* = 0.140 and *P* = 0.001) and short-term warming (*K* = 0.042 and *P* = 0.045), but not for long-term warming (*K* = 0.026 and *P* = 0.444). The growth response (ΔEAF) to short-term warming (λ < 0.001 and *P* = 1, *K* = 0.019 and *P* = 0.580) and long-term warming (λ < 0.001 and *P* = 1, *K* = 0.004 and *P* = 0.806) also lacked significant phylogenetic signal.

**TABLE 1:** Blomberg’s *K* and Pagel’s λ of bacterial ^18^O incorporation in response to warming^*a*^

Treatment/Response	Blomberg’s *K*	*P* value	Pagel’s λ	*P* value
EAF control	0.140	0.001	0.968	<0.001
EAF short-term warming	0.042	0.045	0.943	<0.001
EAF long-term warming	0.026	0.444	0.774	<0.001
ΔEAF short-term warming	0.019	0.580	<0.001	0.999
ΔEAF long-term warming	0.004	0.806	<0.001	0.999

a The table shows values for Blomberg’s *K* and Pagel’s λ of bacterial ^18^O incorporation (i.e., excess atom fraction [EAF]) and change in ^18^O incorporation in response to warming (i.e., ΔEAF).

**FIG 4:**
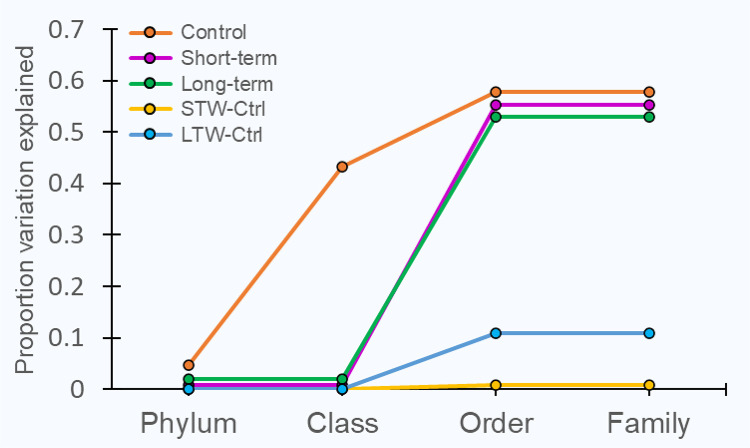
Variance components of ^18^O incorporation (i.e., excess atom fraction; EAF) and change in ^18^O incorporation in response to warming (i.e., ΔEAF) by phylogenetic level. STW, short-term warming; LTW, long-term warming.

The nested taxonomic linear mixed-effect models significantly fit for ^18^O incorporation in control (*P* = 0.014), short-term warming (*P* < 0.001), and long-term warming (*P* < 0.001), as well as the bacterial community growth responses to short-term warming (*P* = 0.006) and long-term warming (*P* < 0.001). Up to 58% of variation of ^18^O incorporation was explained by taxonomy (Fig. 4). The taxonomic level by which the greatest variation in relative growth rate could be explained in the short-term and long-term warming treatment was order (54% and 51%, respectively), but class most explained the variation of relative growth rates in the control (39%). However, the variation of the growth response (ΔEAF) was less attributable to taxonomy according to our model. The long-term warming response was most explained by order (11%), but little variation in the short-term warming response was explained by taxonomy (0.08% of total variation).

**FIG 5:**
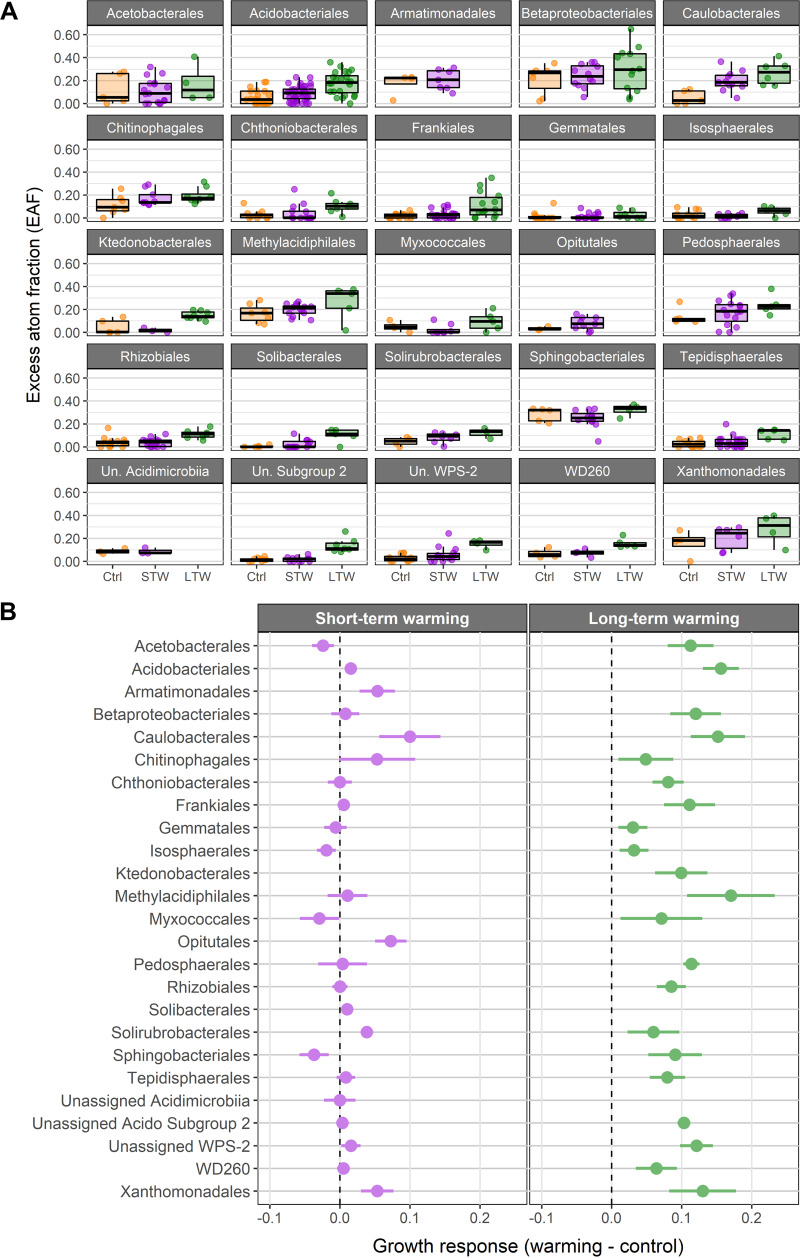
(A) Boxplots of ^18^O incorporation (i.e., excess atom fraction [EAF]) of each taxonomic order by treatment STW, short-term warming; LTW, long-term warming. (B) Mean ^18^O incorporation (i.e., ΔEAF) of bacterial orders in response to short-term and long-term warming. A shift to the right indicates that an order grew more in a warming treatment, while a shift to the left indicates that an order grew less. Only orders with three or more taxa occurring in both a warming treatment and the control are included.

Based on the taxonomic levels by which bacterial growth rate was most constrained, we calculated the aggregate growth responses of bacterial orders to short-term and long-term warming (ΔEAF; Fig. 5). All orders had positive growth responses to long-term warming ranging from 0.02 to 1.7 ΔEAF. In contrast, short-term warming induced both positive and negative growth responses ranging from −0.03 to 1.0 ΔEAF, but short-term warming did not induce a growth response for most bacterial orders. Additionally, boxplots of relative growth rates of each taxonomic phylum (Fig. S2) and class (Fig. S3) are reported for each treatment.

## DISCUSSION

Northern latitudes will be especially affected by climate warming, and carbon flux to the atmosphere by tundra ecosystems will have significant impacts on the future global climate. Resultant changes in microbial activity will ultimately determine the rate of mineralization of soil organic carbon (7), but measurement of the effects of warming on the activity of populations within complex soil microbial communities remains challenging. To better understand the microbial response to climate change in tundra ecosystems, we measured the taxon-specific bacterial growth response to short-term and long-term warming via qSIP field assays, a technique that was limited to laboratory experiments until only recently (35). As predicted, both short-term and long-term warming increased the mean relative growth rates of bacteria (Fig. 1), but the growing bacterial community responded to each warming treatment in distinct ways.

Short-term warming in the tundra increases mineralization of soil organic matter as this process is constrained by temperature. Augmented rates of decomposition can result in increased soil organic carbon mineralization and increased plant growth as stimulated by the increased cycling of nutrients, particularly nitrogen and phosphorus (12). This phenomenon was observed after as little as 1.5 years of warming where soil carbon decreased, plant biomass increased, and functional gene abundances of carbon and nitrogen transformations increased (14). Our results suggest that temperature and any accumulated indirect effects after 3 months of warming selected for many new growing bacteria that perform these metabolic functions (Fig. 2) rather than inducing a warming response of growing bacteria shared with the control (Fig. S1; Fig. 5). The large, emergent cohort of short-term warming responders may represent a transient community that is responsible for the increased microbial activity found to occur after initial warming in soil across systems (52). However, in long-term warming, this transient growing community diminished and was replaced by growing taxa shared with the control (Fig. 2). These findings provide insights into the successional response of tundra bacteria to warming (53, 54) and are also revealing when contextualized within a disturbance-recovery framework (55). Soil microbial communities tend to be sensitive to most disturbances associated with climate change, including warming (56). In the short term, warming substantially altered the composition of growing bacteria due to the emergent taxa, which could be characterized as disturbance-associated taxa. However, a portion of the growing community remained stable in response to warming as the growth rates of taxa shared in all treatments remained unchanged in the short term and then increased in the long term. This could be interpreted as the growing bacterial community exhibiting sensitivity to warming as a disturbance in the short-term while demonstrating resilience by recovering in the long-term (55).

A meta-analysis of soil warming studies concluded that warming elicits the greatest response from the soil microbial community directly after initial warming and attenuates over time in response to decreased available soil carbon, especially at the decade scale (52). Recent field qSIP measurements in montane grassland soil corroborated this by linking decreased prokaryotic growth to depleted soil carbon stocks in response to long-term warming (35). In accordance with the predictions of Romero-Olivares et al. (52), initial warming here also increased the growth rates and diversity of growing taxa (Fig. 1 and 2). However, the tundra bacterial growth response to long-term warming was 7-fold greater than the short-term warming response, and all orders responded positively to long-term warming (Fig. 5), indicating that the growth rates of bacteria continue to increase over time. A concurrent study in these plots reported decreases in total soil carbon with increases in dissolved organic carbon (49). While total soil carbon may be similarly declining in response to long-term warming in these plots, higher bacterial growth rates are likely supported by increased labile substrates allocated belowground by larger deciduous shrubs. Indeed, increased shrub dominance as observed in these plots (24) increases rhizodeposition, which has been shown to stimulate decomposition of old soil organic carbon (57) and facilitate microbial priming of soil organic matter as a means of nitrogen mining (17).

Comparing the magnitude of our observed bacterial growth responses to predicted intrinsic temperature sensitivities can provide insight into to the relative influence that direct effects of warming may have on tundra bacterial activity. The temperature sensitivity of soil microbial activity is determined by physiological constraints of the microbial community (58) and environmental factors such water availability (59, 60) and substrate availability and quality (58). Further, the physiological constraints on microbial activity (i.e., intrinsic temperature sensitivity) are affected by the ambient soil temperatures, with lower temperature soils exhibiting relatively higher intrinsic temperature sensitivities (61). The average soil temperatures in our plots for the duration of the experiment were 0.83°C in the control and 2.3°C in the greenhouse (49), corresponding to an intrinsic temperature sensitivity Q_10_ of approximately 7.25 as predicted by Kirschbaum’s model (61). This closely aligns with our observed temperature sensitivity of bacterial growth to short-term warming (Q_10_ = 6.83), indicating that the magnitude of this response could potentially be explained entirely by the bacterial community’s intrinsic temperature sensitivity. Contrastingly, long-term warming induced such a large response (Q_10_ = 315) that most of the growth response cannot be attributed to intrinsic temperature sensitivity but rather to other environmental factors that increase sensitivity, such as increased inputs belowground (12, 57) and increased recalcitrance of available substrates (13, 58).

In addition to accelerated growth, long-term warming and the subsequent accumulated effects of warming have likely decreased bacterial biomass in these experimental plots as in other tundra habitats (based on unchanging total microbial biomass and increasing fungi:bacteria ratios [12, 62, 63]), suggesting a faster growing bacterial community of reduced size. Further, increasing bacterial growth rates coupled with decreasing size of bacterial populations indicates that long-term warming resulted in higher rates of bacterial turnover. This is consistent with other studies of the influence of warming on microbial biomass turnover rates (64–66), and it may have a significant impact on soil nitrogen dynamics in a warmer tundra because microbial biomass functions as an important nitrogen reservoir in tundra ecosystems (67). Increased biomass turnover will result in increased available nitrogen via microbial necromass accumulation (68, 69), but nutrients derived from dead or lysed bacterial cells can also become vulnerable to leaching (67). Increased bacterial turnover may be a consequential effect of increased temperatures that modulates the mineralization of limiting nutrients such as nitrogen and phosphorus. Conducting qSIP studies exploring the growth responses of other microbial groups (e.g., fungi) and microbial necromass accumulation would further illuminate the effects of warming on elemental cycling via microbial biomass turnover.

We also predicted that bacterial growth rates in tundra soil would exhibit phylogenetic organization. Indeed, bacterial relative growth rates exhibited phylogenetic organization in all treatments, although the phylogenetic signal for growth rates was weaker in the long-term warming treatment (Table 1). Nonetheless, phylogenetic organization of bacterial growth in each treatment was confirmed by the variance component estimates of ^18^O incorporation by taxonomic level where the majority of the variation in relative growth rates could be explained by class and order in all treatments (Fig. 4). Other studies of the phylogenetic organization of bacterial growth found that taxonomic ranks from phylum to family predicted a portion of bacterial growth (45, 47, 70). Here, however, the variation of relative growth rates was explained almost exclusively by both class and order in the control and by order only in the warming treatments. Our results suggest that bacterial growth rates in the tundra when measured in the field are relatively more constrained by deeper evolutionary relationships compared to similar studies of the phylogenetic organization of bacterial growth. The patterns observed here may deviate from previous findings because they are from a different ecosystem. Alternatively, growth rates when measured in the field may more closely represent their realized phenotypes for growth in nature (38), and they may be more deeply phylogenetically conserved compared to qSIP incubations performed in the lab.

Soil bacterial responses to warming have been observed to exhibit phylogenetic organization (36, 71), yet these findings were not corroborated by our results (Table 1; Fig. 4). However, the lack of detection of phylogenetic signal within bacterial growth responses may be because orders shared between the control and warming treatments responded consistently, with a mostly neutral response to short-term warming and a consistently positive response to long-term warming (Fig. 5). Further, aggregating the growth responses by phylogenetic group shows consistent responses within orders as we would expect when growth rates in all treatments are phylogenetically conserved at similar taxonomic levels. Grouping the warming responses by broad taxonomic levels can reduce the complexity of the interpretation of our results, and this is especially informative when considering orders that are more functionally cohesive because we can then extend the interpretation of an order’s warming response to effects on ecosystem processes. For example, the order *Methylacidiphilales* exhibited the greatest growth response to long-term warming, and taxa within this order are putative methanotrophs (72) with few known exceptions (73). Rates of methanogenic archaea and methanotrophic bacteria determine flux of methane, a potent greenhouse gas, from tundra ecosystems to the atmosphere (74). Increased growth rates and implicit rates of methane oxidation of *Methylacidiphilales* are likely supported by increased soil methane concentrations in response to long-term warming (75). Other functionally important orders responded similarly to long-term warming, such as *Rhizobiales* and *Frankiales*, which include nitrogen-fixing bacteria that associate with root nodules of the woody shrubs that dominate warmed tundra (76). We also found that the order *Myxococcales* responded negatively to short-term warming and positively to long-term warming. *Myxococcales* (i.e., myxobacteria) are facultative predators of prokaryotes and other microorganisms (77), suggesting divergent responses of higher trophic levels to short-term and long-term warming (78).

### Conclusion.

Growth rates of soil bacteria were very sensitive to temperature and the accumulated effects of warming in Arctic tundra. Three months of warming of approximately 1.5°C did not induce a response from most bacterial orders but instead stimulated the emergence of a transient cohort of temperature respondent taxa. However, long-term warming supported more than double the growth of the bacterial community and induced a response from all orders. The magnitude of this response far exceeded that which is predicted by intrinsic temperature sensitivity, suggesting that most of the increased bacterial growth in the long-term warming is supported by the accumulated indirect effects of warming (e.g., shrub encroachment, altered litter input abundance and stoichiometry, etc.). Moreover, community-level increases in microbial growth rate could have long-term impacts on nutrient cycling via microbial biomass. We also found that growth of Arctic tundra bacteria is tightly constrained by deep evolutionary relationships. Knowing that over half of the variation in growth rate is explained by broad taxonomic groups may allow for more generalized predictions of the bacterial growth in tundra soils and facilitate the explicit incorporation of soil microorganisms into phylogenetically informed soil models.

## MATERIALS AND METHODS

### Experimental design.

We performed our investigation of the effects of warming on bacterial growth in plots within the Arctic Long-Term Ecological Research (ARC LTER) site at Toolik Lake Research Station, Fairbanks, AK, USA (68° 38' N, 149° 36.4′ W). This site is on a moraine formed by Itkillik I glacial drift and was deglaciated 60,000 years ago (79, 80). Soils at this site are in moist acidic tussock tundra and are classified as poorly drained typic aquaturbels (81). Vegetation is dominated by Betula nana dwarf shrubs and tussock-forming Eriophorum vaginatum sedges (82). Our experiment was conducted entirely during the growing season, which is 50 to 70 days long with a mean temperature of 10°C. The overall mean annual temperature at Toolik Lake is –7°C, and the mean annual precipitation is 400 mm, about half of which is snow (83).

The experimental array includes four plots that have been passively warmed with greenhouses of transparent plastic since 1989 (29 years of warming; see the methods of DeMarco et al. [20]) and four control plots. To achieve short-term warming, intact blocks (20 cm × 20 cm × 30 cm) of tundra plants and soil were transplanted from each control plot to an adjacent greenhouse. To control for the effects of transplant, additional tundra blocks were cut out of each of the control and greenhouse plots and immediately placed back into their plot of origin. All transplants and “mock transplants” were performed in April 2018. Previous studies reported that these greenhouses have increased average summer soil temperatures 1.2 to 2.0°C (63, 84), and an increase of 1.5°C was reported during the summer of our experiment (49).

In July, 3 months after experimental setup, qSIP field assays were performed in each block (four control, four short-term warming, four long-term warming) so that the measurements of bacterial growth would coincide with peak net primary productivity. Parallel assays of natural abundance (^16^O) or enriched (^18^O) water were conducted in each block (24 assays total) in areas of the soil between tussocks. Native soil water was removed via desiccation then added back as ^16^O or ^18^O water as follows. First, the deep organic soil horizon (24, 48) was collected by peeling back a 5-cm layer of vegetation and coring to a depth of 20 cm (3.5-cm core diameter). The mineral layer was discarded, if present, and the organic layer of soil was stored in plastic bags and briefly refrigerated before processing. The mineral layer was never found to be greater than 3 cm thick. The soil in bags was gently manipulated to remove large roots while keeping the soil structure intact. Wet soil (44 g) from each core was divided into 4 tubes made of 42-μm plastic mesh, weighed, and placed into 125-mL Nalgene bottles containing 40 g desiccation beads (10-18 mesh; Fisher Scientific lot 171721) and desiccated for 24 h at ambient temperature. Subsamples of wet soils and desiccated soils were oven dried at 60°C for 72 h to measure field and postdesiccation soil water content. The average water content of soils collected from the field was 81.6%, and the average water content after desiccation was 56.7%. Three grams of partially dried soil from each block were placed in 15-mL Falcon tubes, and then the soil was restored to field conditions by injecting 3.3 mL ^16^O or ^18^O (98 atm %) water into the soil. The average final water enrichment of ^18^O incubations was 66 atm %. The headspaces of the Falcon tubes were filled with glass wool, and then the tubes were sealed with parafilm, allowing gas exchange while preventing water contamination. Sealed Falcon tubes were placed vertically in the block of origin at depths between 10 and 20 cm. After 30 days, the tubes were collected and placed in a cooler with dry ice, shipped to Northern Arizona University, and stored frozen at –80°C for molecular analysis.

### DNA extraction, qSIP fractionation, and sequencing.

The soil samples were processed according to Purcell et al. (85). Briefly, DNA was extracted and quantified, and then 1.0 to 1.5 μg of DNA from each sample was separated via isopycnic ultracentrifugation in saturated cesium chloride. Ultracentrifuged DNA was separated into density fractions and then purified via isopropanol precipitation. Prokaryotic 16S rRNA gene copy numbers of all fractions were measured using quantitative PCR (qPCR). Fractions with detectable 16S copies were sequenced using on an Illumina MiSeq at the Genetics Core Facility at Northern Arizona University. We prepared DNA for sequencing according to Fadrosh et al. (86) using the updated Earth Microbiome Project primers 515F (5′-GTGYCAGCMGCCGCGGTAA-3′ [87]) and 806R (5′-GGACTACNVGGGTWTCTAAT-3′ [88]) for 16S amplification and index PCR.

### Sequence analysis and calculations of stable isotope incorporation.

Sequences were quality filtered and analyzed using QIIME2 (89). The QIIME2 pipeline for sequence analysis was previously described by Purcell et al. (85). Briefly, the sequences were demultiplexed and then corrected and filtered with DADA2 (90). Denoised sequences were left as amplicon sequence variants (ASVs) rather than clustered as operational taxonomic units (OTUs) (91). Taxonomy was assigned to ASVs using the q2-feature-classifer plugin with a pretrained, naive Bayes classifier for the SILVA database (92) of the 515F/806R 16S region (silva-132-99-515-806-nb-classifier-2019.4.qza [93]).

Relative abundance tables of ASVs with assigned taxonomies were then exported for qSIP calculations according to Finley et al. (94). Calculations, filtering, subsequent analyses, and figure creation were performed in R (95). For each pair of ^16^O and ^18^O incubations in each treatment, the change in weighted average density (WAD) of each taxon was calculated using its relative abundance (sequences) and 16S gene copy number (qPCR) of each density fraction (49). The excess atom fraction (EAF) ^18^O incorporated into a taxon’s DNA was determined based on the shift in WAD in the ^18^O incubation compared to the parallel ^16^O incubation. As a filtering step, EAF was calculated only for ASVs that occurred in three or more consecutive fractions (77), and taxa for which the initial estimate of ^18^O EAF was less than zero were assigned an EAF of zero for downstream analyses.

### Data analysis.

Means across treatments were compared via one-way analysis of variation (ANOVA) and Tukey’s HSD *post hoc* test. Student’s *t* tests were used when comparing means that occurred in only two treatments (e.g., mean EAF of taxa shared with the control and long-term warming). Taxon-specific responses of warming treatments (i.e., EAF_treatment_ – EAF_control_) were calculated, here referred to as ΔEAF. Temperature sensitivities expressed as Q_10_, a metric defined as the fold increase of a process in response to a temperature increase of 10°C, were also calculated using the van’t Hoff equation (58, 61). For warming responses aggregated at broader phylogenetic levels, the analysis included taxonomic groups with three or more representatives in both the warming treatment and the control.

The phylogenetic organization of EAF and ΔEAF were assessed similarly to the methods of Morrissey et al. (43). Blomberg’s *K* and Pagel’s λ were used as indices of phylogenetic signals of EAF and ΔEAF (96, 97). First, a phylogenetic tree of all observed ASVs was constructed using PICRUSt2 in Python (98), which uses an insertion tree approach (99). EAFs of each ASV from a treatment were then matched to their corresponding branch tip of the tree; ASVs that did not have measurable ^18^O incorporation were excluded. Blomberg’s *K* and Pagel’s λ were calculated using the phytools R package (100). For the phylogenetic signal analysis of ΔEAF, only ASVs that occurred in both the treatment (short-term or long-term warming) and the control were included. Linear mixed-effects models were also constructed with taxonomic levels as nested random effects to estimate the variance in growth explained by each taxonomic level. Variance components of each level were estimated using the ape package (101).

### Data availability.

The 16S rRNA amplicon sequence data are available at the NCBI Sequence Read Archive under the accession number PRJNA866660 (the sequences can be accessed and downloaded: SRR20883373 and SRR20883374) (102).
