# Supplementary material for: Distinct Growth Responses of Tundra Soil Bacteria to Short-Term and Long-Term Warming
Source: Appl Environ Microbiol. 2023 Feb 27;89(3):e01543-22. doi: 10.1128/aem.01543-22 (PMC10056963; doi:10.1128/aem.01543-22)
Supplement: Supplemental file 1 — Supplemental material. Download aem.01543-22-s0001.pdf, PDF file, 0.2 MB [file aem.01543-22-s0001.pdf]

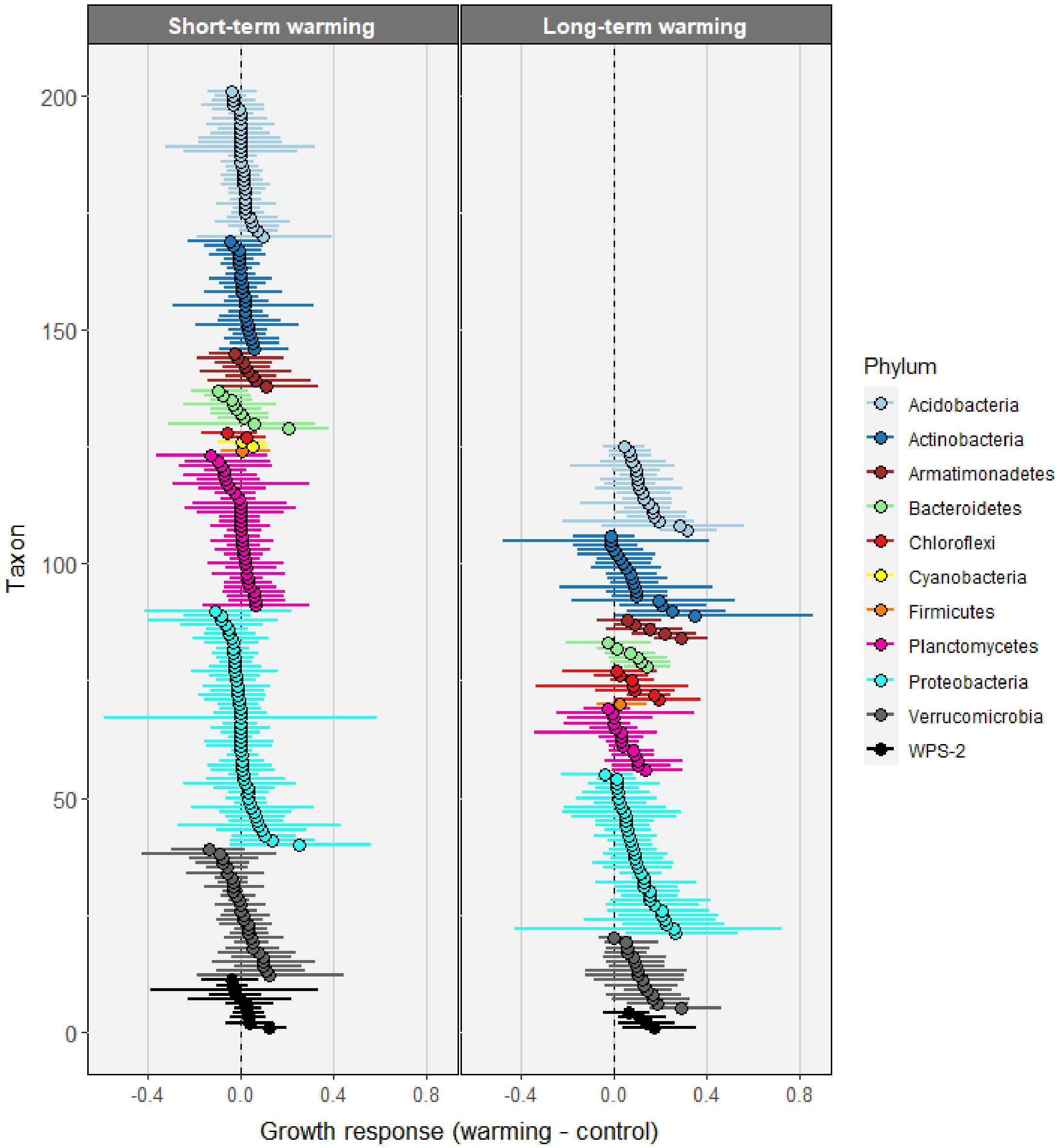

Supplemental Figure 1. Change in ^{18}O incorporation (i.e., ΔEAF) of individual taxa in response to short term and long-term warming. A shift to the right indicates that a taxon grew more in a warming treatment, while a shift to the left indicates that a taxon grew less. Each taxon is colored by phylum, and bars represent the 90% confidence interval of the response.

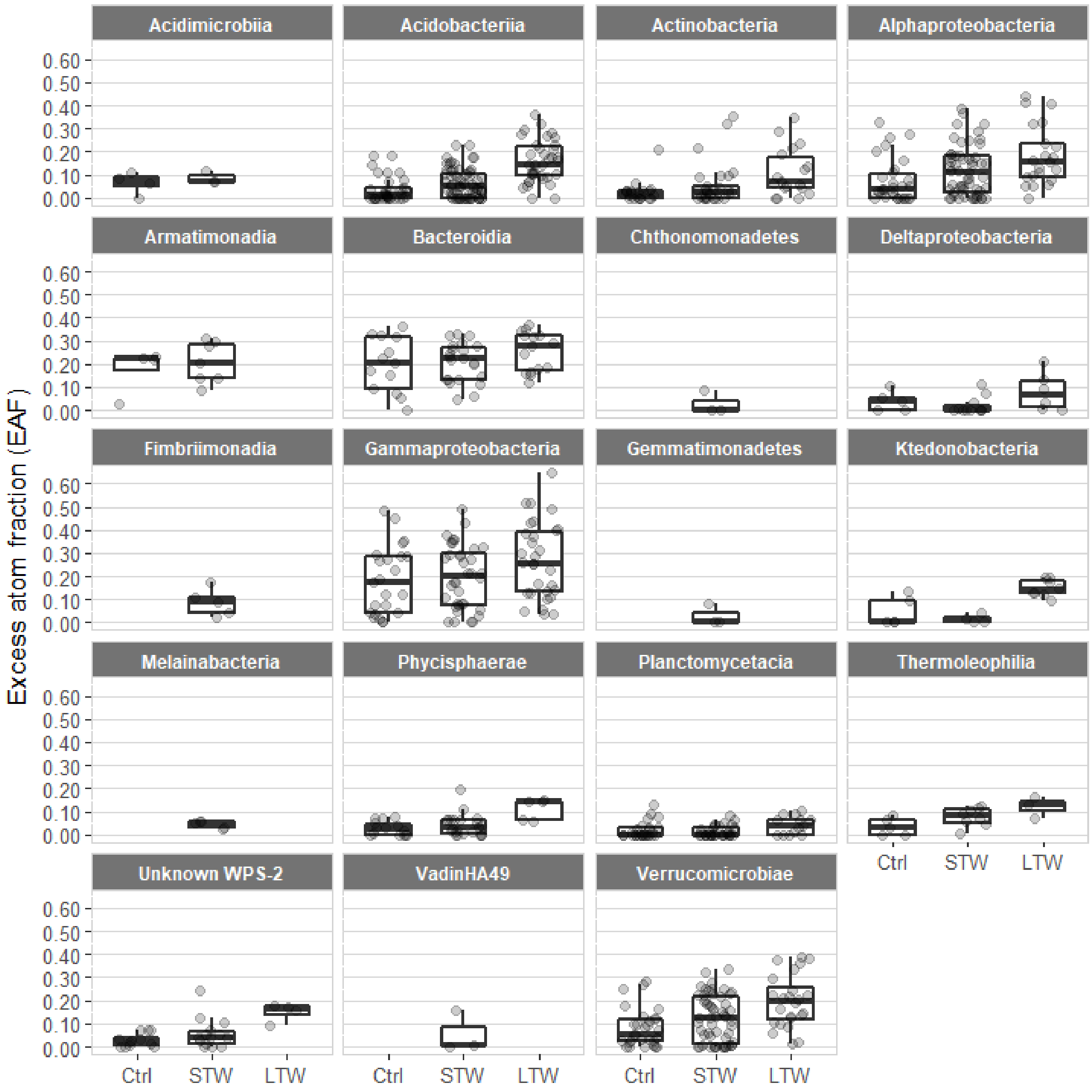

Supplemental Figure 2. Box plots of ^{18}O incorporation (i.e., excess atom fraction; EAF) of each taxonomic class by treatment. Only classes with three of more taxa occurring in a treatment are included.

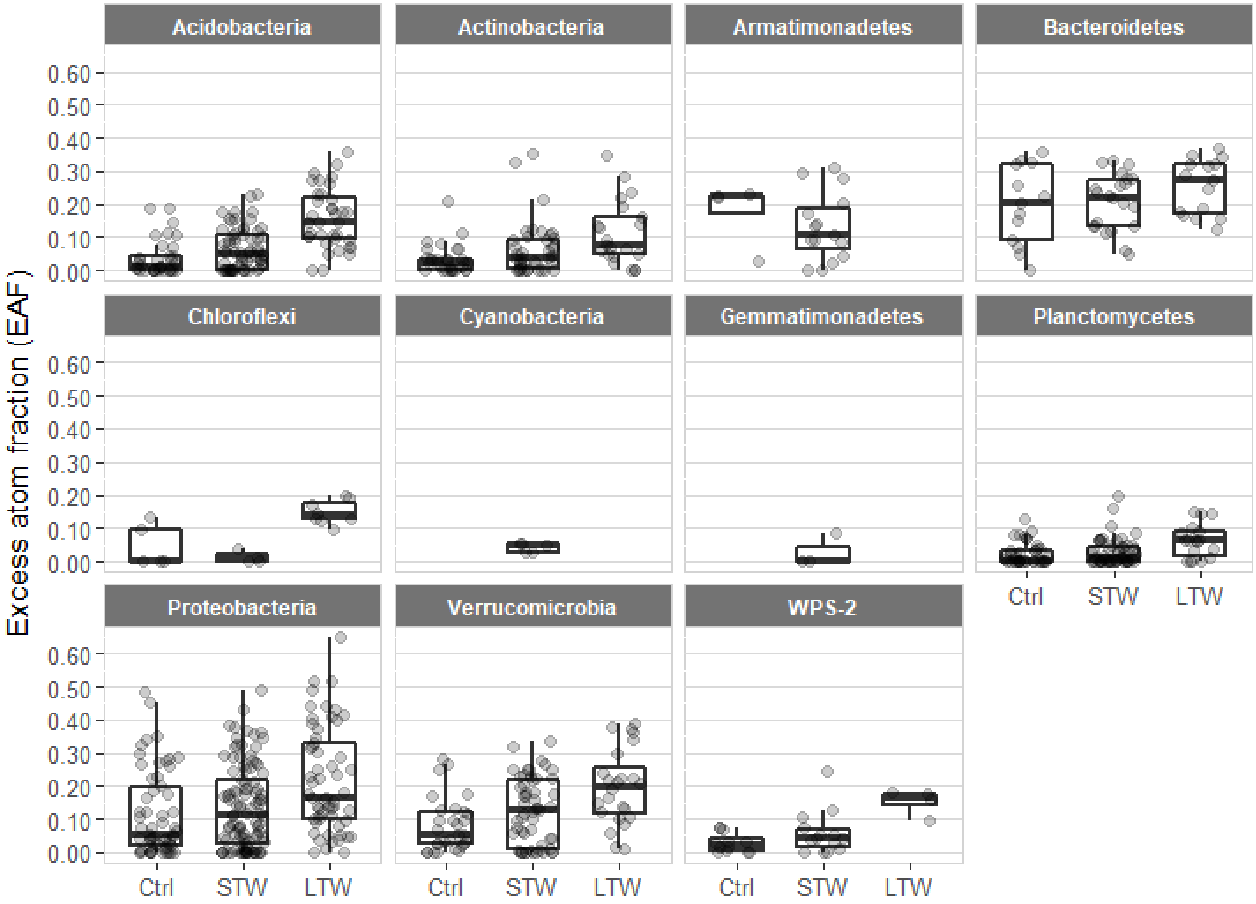

Supplemental Figure 3. Box plots of ^{18}O incorporation (i.e., excess atom fraction; EAF) of each taxonomic phylum by treatment. Only phyla with three of more taxa occurring in a treatment are included.
